# Relationship between duration of intraoperative hypotension and postoperative delirium in patients undergoing head and neck cancer surgery with free flap reconstruction: a retrospective observational study

**DOI:** 10.1007/s00540-025-03538-2

**Published:** 2025-07-13

**Authors:** Norihiko Obata, Daichi Fujimoto, Satoshi Mizobuchi

**Affiliations:** Department of Anesthesiology, Kobe University Hospital, 7-5-1 Kusunoki-Cho, Chuo-Ku, Kobe, Hyogo 650-0017 Japan

**Keywords:** Postoperative delirium, Elderly, Head and neck cancer surgery, Free flap reconstruction, Intraoperative hypotension

## Abstract

**Purpose:**

Postoperative delirium (POD) is a frequent complication after surgery, especially in elderly patients undergoing head and neck cancer surgery with free flap reconstruction. This study aimed to assess the associations between intraoperative hypotension (IOH), its duration, and occurrence of POD.

**Methods:**

This retrospective study included 239 patients aged 65 years or older who underwent head and neck cancer surgery with free flap reconstruction. IOH was defined at seven mean arterial pressure (MAP) thresholds, ranging from 55 to 85 mmHg, in 5 mmHg increments. The duration of each IOH was compared between patients with or without POD before and after initiation of microsurgery. Multivariate analysis was conducted to assess the independent association of each IOH duration with the risk of POD.

**Results:**

POD occurred in 43 (18.0%) of the 239 patients. Before the initiation of microsurgery, the cumulative duration of hypotension below MAP thresholds of < 70 to 80 mmHg was significantly longer in patients with POD. After the initiation of microsurgery, the cumulative duration of hypotension below MAP thresholds of < 55 to 85 mmHg was also significantly longer in patients with POD. In multivariate analysis, the cumulative duration of hypotension below MAP thresholds of 70, 75, and 80 mmHg before and after the initiation of microsurgery was independently associated with POD (*p* < 0.05 at each threshold).

**Conclusion:**

Prolonged IOH, particularly below MAP thresholds of 70, 75, and 80 mmHg, was significantly associated with POD in elderly patients undergoing head and neck cancer surgery with free flap reconstruction.

**Supplementary Information:**

The online version contains supplementary material available at 10.1007/s00540-025-03538-2.

## Introduction

The occurrence of postoperative delirium (POD) is not only associated with an increased duration of hospital stay, greater medical costs, and an increased incidence of complications during hospitalization (including falls, pressure sores, etc.) [1] but can also lead to cognitive decline after discharge from hospital [2] and reduce the chances of returning to normal social activities. Patients undergoing head and neck cancer surgery with free flap reconstruction are considered a high-risk group for POD due to various factors such as preoperative malnutrition, alcohol abuse, and the prolonged duration of the surgery [3]. Preventing the occurrence of POD is important in this patient group. Longer durations of intraoperative mean arterial pressure (MAP) below 55 mmHg or 65 mmHg in non-cardiac surgery have been reported to be significantly associated with a higher incidence of POD [4, 5].

The procedure for head and neck cancer surgery with free flap reconstruction is performed in the following order: tumor resection, free flap harvesting, and reconstruction including microvascular anastomosis of the free flap using a microscope [6]. In other words, the entire surgery can be divided into phases before and after the initiation of microsurgery. In free flap surgery, it has been reported that an intraoperative MAP below 60 mmHg significantly increases the risk of flap failure [7]. Administration of a vasopressor and fluid loading are used to improve blood flow to the free flap [8]. Therefore, in free flap surgery, the intraoperative MAP is maintained at a higher level than that typically in common surgeries [9].

There has been only one study in which the relationship between intraoperative blood pressure and POD in flap surgeries was examined. In that study, the cutoff value for the lowest intraoperative MAP associated with POD was found to be 62.5 mmHg [10]. However, the duration of hypotension was not examined in that study. Consequently, there has been no report on the duration of hypotension during free flap surgery. Therefore, this study was carried out to assess the relationships between intraoperative hypotension (IOH), its duration, and POD in patients undergoing head and neck cancer surgery with free flap reconstruction.

## Methods

Study design: This single-center, retrospective observational study was approved by the ethics committee of Kobe University Hospital (Approval No. B240063). The requirement for informed consent was waived due to the study’s retrospective design. The data used in this study were anonymized to protect patient privacy. Ethical considerations regarding the retrospective nature of the study and the waiver of informed consent were reviewed and approved by the ethics committee of Kobe University Hospital. We collected data using the hospital's electronic medical records system for patients aged 65 years or older who underwent head and neck cancer surgery with free flap reconstruction at Kobe University Hospital between January 2015 and December 2023. Patients with incomplete data, such as missing postoperative Intensive Care Delirium Screening Checklist (ICDSC) or Critical Care Pain Observation Tool (CPOT) scores within 2 days after surgery and missing information for the timing of microsurgery initiation, and patient who underwent a reoperation within 2 days after surgery were excluded.

Standard anesthesia protocol: Premedication was not administered to any patients preoperatively. After initiating inhalation of 100% oxygen, remifentanil infusion was started at 0.2–0.5 μg/kg/min, followed by induction of anesthesia with propofol at 1–2 mg/kg. After confirming loss of consciousness, rocuronium was administered at 0.6–0.8 mg/kg for endotracheal intubation. Tracheostomy was performed first, followed by the commencement of surgery. In cases in which airway management risk was deemed high, tracheostomy under local anesthesia was performed before the induction of general anesthesia. General anesthesia included remifentanil at 0.1–0.5 μg/kg/min, along with maintenance of propofol at 3–5 μg/ml using a TCI pump, or inhalational agents such as desflurane at 3–5% or sevoflurane at 1–1.5%, with intermittent use of fentanyl and a vasopressor (ephedrine or phenylephrine) as needed. During free flap reconstruction, blood pressure was managed at the discretion of the attending anesthesiologist, potentially using dopamine, dobutamine, or noradrenaline. Postoperatively, most patients were returned to the ICU under sedation with propofol, fentanyl, and dexmedetomidine.

## Collected data

### We collected the following perioperative data from medical records.

Basic characteristics: We obtained information for patient characteristics including age, sex, height, body weight, body mass index (BMI), and American Society of Anesthesiologists physical status (ASA-PS). We also obtained information for the presence of complications such as cognitive impairment, depression, schizophrenia, Parkinson’s disease, cerebrovascular diseases, ischemic heart disease, valvular disease, hypertension, asthma, chronic obstructive pulmonary disease (COPD), restrictive lung disease, diabetes, hemodialysis, preoperative benzodiazepine use, alcohol abuse, and smoking history. We also obtained information on results preoperative blood examination including sodium, potassium, chloride, albumin, blood urea nitrogen (BUN), creatinine, estimated glomerular filtration rate (eGFR).

### Intraoperative factors

We obtained information for operation time, anesthesia method (inhalation anesthesia or total intravenous anesthesia), use of epidural anesthesia, doses of remifentanil and fentanyl, ketamine, dexmedetomidine, vasopressor agents (ephedrine, phenylephrine, dopamine, dobutamine, and noradrenaline), phosphodiesterase (PDE)3 inhibitors, and analgesic agents (acetaminophen and flurbiprofen). We also obtained information for blood transfusion, blood loss, fluid infusion, and urine output. Additionally, we obtained operation information including operation time, neck dissection, presence of tracheostomy, harvesting site of the free flap (jejunal, rectus abdominis, fibula, radial forearm), and intraoperative data per 1 min (heart rate, SpO_2_, temperature, arterial blood pressure [systolic, mean, diastolic]).

Postoperative data: Postoperative information obtained included information on discharge from the operating room with or without sedation, use of fentanyl, dexmedetomidine, and haloperidol in the ICU, assessments of CPOT and ICDSC scores during the first two postoperative days, and the lengths of ICU stay and hospital stay.

### Definition of IOH

Intraoperative arterial pressures were recorded every minute for all patients. We considered the following values as artifacts and excluded blood pressure data when these values were present before analysis: (1) systolic blood pressure > 250 mmHg or < 30 mmHg, (2) mean blood pressure > 200 mmHg or < 25 mmHg, (3) diastolic blood pressure > 100 mmHg or < 10 mmHg, and (4) systolic blood pressure > mean arterial pressure > diastolic blood pressure sequence not maintained.

Since there is no single threshold for defining IOH, we predefined 7 different absolute thresholds of IOH as follows: mean blood pressure < 55 mmHg, < 60 mmHg, < 65 mmHg, < 70 mmHg, < 75 mmHg, < 80 mmHg, and < 85 mmHg.

In free flap surgery, blood pressure should be maintained at a normal level or slightly elevated level after the initiation of microsurgery to maintain flap perfusion [9]. Therefore, we investigated the relationship between intraoperative hypotension and POD during the following two periods: (1) before the initiation of microsurgery and (2) after the initiation of microsurgery. We then investigated the relationship between the cumulative duration below the above-mentioned 7 thresholds during these two periods and POD.

### Primary outcome

The primary outcome of this study was the occurrence of delirium within 2 days after surgery. Delirium diagnosis was based on the ICDSC with a threshold set at 4 points or higher in at least one of the ICDSC assessments during a 48-h period after surgery. In our ICU, ICDSC scoring is conducted every 8 h by nurses.

### Statistical analysis

The patients were divided into two groups based on the occurrence of POD (patients with POD: delirium group, patients without POD: no delirium group), and the two groups were compared. Categorical data are presented as total numbers (%) and they were analyzed using the chi-square test. Continuous data are presented as medians (interquartile range) and they were analyzed using the Mann–Whitney U test. We compared IOH values in patients with and those without POD using the Mann–Whitney U test.

Multivariate logistic regression analysis was conducted to adjust for associations with other confounding factors related to the occurrence of POD. Given that a robust multivariate model typically requires 5 to 9 events per predictor variable, we estimated that our dataset would allow for the inclusion of 5 to 9 variables in the analysis [11]. Confounding factors were selected on the basis of their strong association with POD, as in previous studies, or according to the results of univariate analysis: age, sex, smoking history, pre-existing cognitive impairment, albumin, blood transfusion, average CPOT score within two days in the ICU, and hypertension [12, 13].

The odds ratio for the duration of hypotension was calculated as the odds ratio per hour.

This study included the total number of patients available during a 9-year period. As this is an exploratory study, no sample size calculation was conducted. Statistical analyses were performed using the statistical program EZR [14]. A p value of < 0.05 was considered to indicate a statistically significant difference. Standardized mean difference(SMD) was presented for all data.

## Results

### Study flow (Fig. 1)

**Fig. 1:**
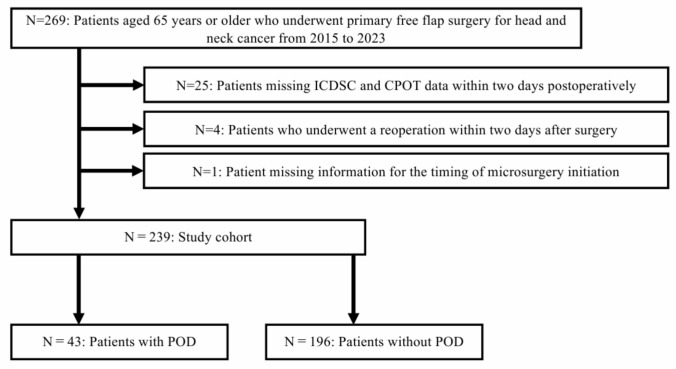
Study flow

During the observation period, 269 patients aged 65 years or older underwent head and neck cancer surgery with free flap reconstruction. Among them, 25 patients without ICDSC data and CPOT data within 48 h after surgery, 4 patients who underwent a reoperation within 48 h after surgery, and 1 patient with unknown initiation time of microscopic surgery were excluded. The final study cohort included 239 patients, among whom 43 (18.0%) developed POD within 48 h after surgery.

### Comparison of the characteristics of patients in the delirium group and no delirium group

Table 1 shows the preoperative characteristics of the patients. There was no significant difference between the two groups except for age and preoperative cognitive impairment. Patients in the delirium group were significantly older (*p* = 0.031) and had a significantly higher prevalence of preoperative cognitive impairment (*p* = 0.011). Table 2 shows the intraoperative and postoperative characteristics of the patients. There was no significant difference between the two groups except for intraoperative blood pressure. The delirium group had significantly lower levels of systolic blood pressure (*p* = 0.003), mean arterial pressure (*p* = 0.001), and diastolic blood pressure (*p* < 0.001).

**Table 1:** Preoperative patients baseline characteristics

	No delirium (n = 196)	Delirium (n = 43)	*p*-value	SMD
Characteristics				
Age (years)	73.5 [70.0, 78.0]	77.0 [71.5, 81.0]	0.03	0.378
Sex				
Male (%)	136 (69.4)	27 (62.8)	0.47	0.14
Female (%)	60 (30.6)	16 (37.2)		
Height (cm)	161.9 [155.0, 166.2]	158.8 [151.8, 164.8]	0.2	0.195
Body weight (kg)	55.7 [48.7, 63.8]	52.8 [48.4, 61.3]	0.4	0.142
BMI (kg/m^2^)	21.6 [19.2, 24.1]	21.3 [19.4, 23.1]	0.83	0.025
ASA-PS (%)				
I	12 (6.1)	2 (4.7)	0.57	0.163
II	170 (86.7)	36 (83.7)		
III	14 (7.1)	5 (11.6)		
Past medical history				
Cognitive impairment (%)	2 (1.0)	4 (9.3)	0.01	0.381
Depression (%)	0 (0)	0 (0)	1	< 0.001
Schizophrenia (%)	0 (0)	0 (0)	1	< 0.001
Parkinson disease (%)	2 (1.0)	1 (2.3)	0.45	0.102
Cerebrovascular disease (%)	16 (8.2)	4 (9.3)	0.77	0.04
Ischemic heart disease (%)	9 (4.6)	2 (4.7)	1	0.003
Valvular disease (%)	3 (1.5)	1 (2.3)	0.55	0.058
Hypertension (%)	92 (46.9)	26 (60.5)	0.13	0.274
Asthma (%)	3 (1.5)	1 (2.3)	0.55	0.058
COPD (%)	14 (7.1)	4 (9.3)	0.54	0.079
Restrictive lung disease (%)	5 (2.6)	3 (7.0)	0.16	0.209
Diabetes (%)	45 (23.0)	12 (27.9)	0.55	0.114
Hemodialysis (%)	0 (0)	0 (0)	1	< 0.001
Preoperative use of benzodiazepines (%)	10 (5.1)	1 (2.3)	0.69	0.147
Alcohol abuse (%)	3 (1.5)	0 (0)	1	0.176
History of smoking (%)	115 (58.7)	27 (62.8)	0.73	0.084
Blood examinations				
Na (mmol/L)	140.0 [137.0, 141.0]	140.0 [137.5, 141.0]	0.7	0.118
K (mmol/L)	4.3 [4.0, 4.5]	4.2 [4.0, 4.5]	0.81	0.006
Ca (mmol/L)	9.1 [8.8, 9.5]	9.1 [9.0, 9.4]	0.99	0.071
Cl (mmol/L)	105.0 [103.0, 106.3]	104.0 [103.0, 106.5]	0.88	0.017
Albumin (g/dL)	3.8 [3.5, 4.2]	3.7 [3.4, 4.2]	0.32	0.258
BUN (mg/dL)	15.7 [12.6, 19.5]	15.5 [11.9, 19.5]	0.96	0.028
Creatinine (mg/dL)	0.81 [0.66, 0.96]	0.79 [0.68, 0.98]	0.78	0.186
eGFR (ml/min/1.73m^2^)	65.4 [56.1, 76.7]	64.2 [53.7, 75.2]	0.32	0.249

The data are presented as medians [25%, 75% interquartile range] or numbers (percentage)

*BMI* body mass index, *ASA-PS* American Society of Anesthesiologists physical status, *COPD* chronic obstructive pulmonary disease, *BUN* blood urea nitrogen, *eGFR* estimated glomerular filtration rate, *SMD* standardized mean difference

**Table 2:** Intraoperative and postoperative information

	No delirium (n = 196)	Delirium (n = 43)	*p*-value	SMD
Intraoperative factors				
Operation time (min)	644.5 [572.5, 714.5]	648.0 [600.0, 721.5]	0.74	0.068
Type of Anesthesia method				
Inhalation anesthetics (%)	190 (96.9)	42 (97.7)	1	0.045
Intravenous anesthetics (%)	6 (3.1)	1 (2.3)		
Use of Epidural anesthesia (%)	6 (3.1)	0 (0.0)	0.6	0.251
Dose of remifentanil (μg/kg/min)	0.18 [0.15, 0.22]	0.18 [0.14, 0.22]	0.52	0.199
Dose of fentanyl (μg)	1000.0 [750.0, 1250.0]	1000.0 [800.0, 1250.0]	0.99	0.038
Ketamine (%)	0 (0)	0 (0)	1	< 0.001
Dexmedetomidine (%)	185 (94.4)	39 (90.7)	0.49	0.141
Vasopressor agents				
Ephedrine (mg)	24.0 [12.0, 36.0]	24.0 [12.0, 36.0]	0.94	0.024
Phenylephrine (mg)	0.00 [0.00, 0.50]	0.25 [0.00, 0.65]	0.18	0.089
Dopamine (%)	57 (29.1)	15 (34.9)	0.47	0.125
Dobutamine (%)	2 (1)	0 (0)	1	0.144
Noradrenalin (%)	21 (10.7)	6 (14.0)	0.59	0.099
PDE3 inhibitor (%)	1 (0.5)	0 (0.0)	1	0.101
Analgesic agents				
Flurbiprofen (%)	6 (3.1)	1 (2.3)	1	0.045
Acetaminophen (%)	7 (3.6)	2 (4.7)	0.67	0.054
Blood transfusion (%)	39 (19.9)	14 (32.6)	0.1	0.291
Blood loss (ml)	435.0 [320.0, 694.0]	506.0 [360.0, 735.0]	0.2	0.193
Fluid infusion (ml)	5200.0 [4322.5, 6077.5]	4950.0 [3925.0, 5825.0]	0.29	0.184
Urine output (ml)	1037.5 [564.8, 1680.0]	1000.0 [632.5, 1386.0]	0.49	0.276
Operation information				
Neck dissection (%)	185 (94.4)	40 (93.0)	0.72	0.056
Tracheotomy (%)	139 (70.9)	26 (60.5)	0.2	0.222
Harvesting site				
Jejunal (%)	64 (32.7)	10 (23.3)	0.32	0.31
Rectus abdominis (%)	41 (20.9)	14 (32.6)		
Fibula (%)	23 (11.7)	6 (14.0)		
Radial forearm (%)	68 (34.7)	13 (30.2)		
Vital signs				
Heart rate (bpm)	72.1 [65.8, 80.2]	76.4 [66.5, 82.2]	0.25	0.18
SpO_2_ (%)	99.7 [99.3, 100.0]	99.8 [99.5, 100.0]	0.27	0.061
Body temperature (℃)	37.0 [36.7, 37.2]	37.0 [36.7, 37.3]	0.51	0.133
Systolic blood pressure (mmHg)	109.5 [104.7, 114.0]	106.42 [102.9, 108.7]	0.003	0.497
Mean arterial pressure (mmHg)	71.4 [67.1, 75.3]	68.7 [66.0, 70.6]	0.001	0.599
Diastolic blood pressure (mmHg)	51.5 [47.6, 55.5]	47.9 [46.0, 51.1]	< 0.001	0.575
Postoperative factors				
Sedated discharge from operating room (%)	189 (96.4)	41 (95.3)	0.67	0.054
Use of fentanyl in ICU (%)	187 (95.4)	41 (95.3)	1	0.003
Use of dexmedetomidine in ICU (%)	192 (98.0)	43 (100.0)	1	0.204
Use of haloperidol in ICU (%)	16 (8.2)	7 (16.3)	0.15	0.25
CPOT				
Day 0	0.00 [0.00, 0.21]	0.00 [0.00, 0.32]	0.26	0.246
Day 1	0.00 [0.00, 0.11]	0.00 [0.00, 0.27]	0.28	0.313
CPOT average in 2 days	0.00 [0.00, 0.19]	0.05 [0.00, 0.33]	0.35	0.32
Length of ICU stay (day)	3.0 [2.0, 3.0]	3.0 [2.0, 3.0]	0.25	0.153
Length of hospital stay (day)	42.5 [31.0, 65.5]	47.0 [37.0, 67.0]	0.22	0.041

The data are presented as medians [25%, 75% interquartile range] or numbers (percentage)

*PDE* phosphodiesterase, *CPOT* Critical Care Pain Observation Tool, *ICU* Intensive care unit, *SMD* standardized mean difference

### Relationship between mean blood pressure and POD

Table 3 shows the relationship between mean blood pressure and POD. The MAP was significantly lower in the delirium group than in the no delirium group before the initiation of microsurgery and after the initiation of microsurgery (before initiation of microsurgery: delirium group 68.1 mmHg [65.0–70.8] vs no delirium group 70.4 mmHg [66.2–74.9] *p* = 0.0082), after initiation of microsurgery: delirium group 68.9 mmHg [66.0–71.2] vs no delirium group 72.3 mmHg [68.5–77.0] *p* = 0.00038).

**Table 3:** Mean arterial pressure data before and after initiation of microsurgery

	No delirium (n = 196)	Delirium (n = 43)	*p*-value	SMD
Before initiation of microsurgery				
Max	108 [99, 116]	103 [95.5, 114.5]	0.25	0.09
Mean	70.4 [66.2, 74.9]	68.1 [65.0, 70.8]	0.008	0.497
Min	49 [44.8, 53]	48 [45, 51]	0.72	0.011
After initiation of microsurgery				
Max	101.5 [93, 112]	97 [88, 104]	0.008	0.401
Mean	72.3 [68.5, 77.0]	68.9 [66.0 71.2]	0.0004	0.607
Min	56 [51, 61]	53 [50, 56.5]	0.01	0.462

The data are presented as medians [25%, 75% interquartile range]

*SMD* standardized mean difference

Comparison of the cumulative durations of hypotension at each threshold in the two groups during each period.

**Fig. 2:**
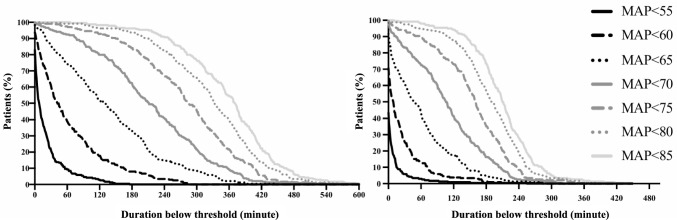
The proportion of patients with hypotension (MAP below the 7 thresholds). The figure on the left shows the proportion of patients with hypotension before initiation of microsurgery. The figure on the right shows the proportion of patients with hypotension after initiation of microsurgery. Each curve corresponds to a different MAP threshold, ranging from < 55 mmHg to < 85 mmHg

**Fig. 3:**
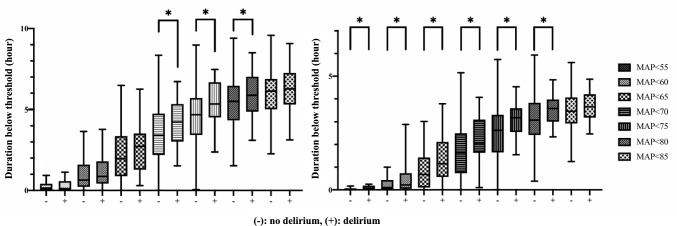
Cumulative duration (median value with interquartile range) below the 7 thresholds. The figure on the left shows the cumulative duration of hypotension before initiation of microsurgery. The figure on the right shows the cumulative duration of hypotension after initiation of microsurgery. Minus (−) indicates patients without POD and plus ( +) indicates patients with POD. Each box plot corresponds to a different MAP threshold, ranging from < 55 mmHg to < 85 mmHg. Asterisk indicates significant difference (p < 0.05)

Figure 2 shows the proportion of patients with hypotension (MAP below the 7 thresholds) and cumulative minutes under the thresholds in each period. Figure 3 and Table 4 show the cumulative durations below the 7 thresholds during each period in the delirium and no delirium groups. During the period before the initiation of microsurgery, the delirium group had a significantly longer duration below each threshold from < 70 to < 80 than did the no delirium group (*p* = 0.01, 0.01, and 0.04, respectively). Additionally, during the period after the initiation of microsurgery, the delirium group had a significantly longer duration below each threshold from < 55 to < 80 than did the no delirium group (*p* = 0.02, 0.02, 0.01, 0.004, 0.003, and 0.01, respectively).

**Table 4:** Duration under each value of threshold (minutes) before and after initiation of microsurgery

	No delirium (n = 196)	Delirium (n = 43)	*p*-value	SMD
Before initiation of microsurgery				
< 55	9.0 [1.0, 24.3]	7.00 [2.0, 34.5]	0.28	0.249
< 60	38.5 [13.0, 95.3]	52.0 [25.0, 107.0]	0.19	0.208
< 65	118.0 [53.0, 201.3]	164.0 [79.5, 209.5]	0.06	0.311
< 70	204.5 [132.5, 283.0]	254.0 [185.5, 319.0]	0.01	0.522
< 75	281.00 [207.3, 342.0]	320.0 [271.5, 389.5]	0.01	0.515
< 80	329.5 [260.8, 386.5]	352.0 [301.5, 418.5]	0.04	0.429
< 85	368.0 [300.8, 412.3]	376.0 [327.0, 435.0]	0.18	0.328
After initiation of microsurgery				
< 55	0.0 [0.0, 4.0]	2.0 [0.0, 9.0]	0.02	0.038
< 60	6.0 [0.0, 26.0]	13.0 [3.0, 43.5]	0.02	0.216
< 65	41.0 [6.8, 85.3]	69.0 [35.5, 120.0]	0.01	0.333
< 70	99.0 [44.0, 150.0]	124.0 [99.0, 182.0]	0.004	0.464
< 75	157.5 [99.0, 198.3]	191.0 [153.5, 213.5]	0.003	0.527
< 80	184.5 [145.0, 228.5]	214.0 [180.5, 235.0]	0.01	0.431
< 85	208.0 [175.8, 243.3]	219.0 [194.0, 251.0]	0.08	0.294

The data are presented as medians [25%, 75% interquartile range]

*SMD* standardized mean difference

### Multivariate analysis

For each IOH value, we adjusted for other factors associated with delirium and investigated whether the hourly duration of hypotension before and after the initiation of microscopic surgery was independently associated with the occurrence of POD.

**Fig. 4:**
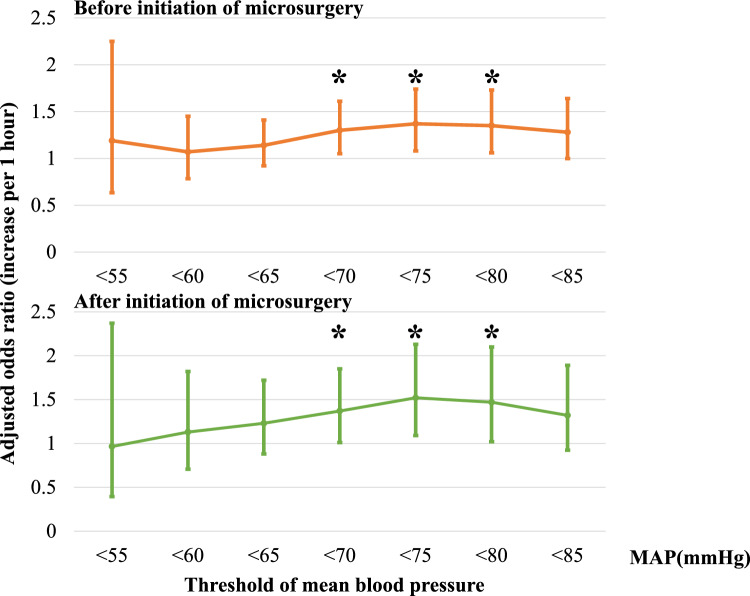
Adjusted odds ratios of POD before and after the initiation of microsurgery for 7 thresholds of hypotension using multivariate analysis. The upper figure shows adjusted odds ratio of POD (increase per 1 h) before initiation of microsurgery. The lower figure shows adjusted odds ratio of POD (increase per 1 h) after initiation of microsurgery. Asterisk indicates significant difference (p < 0.05)

As shown in Fig. 4, before the initiation of microscopic surgery, values of MAP < 70 mmHg (increase per 1 h) (adjusted OR [95%CI] = 1.3 [1.05–1.61], *p* = 0.016), < 75 mmHg (increase per 1 h) (adjusted OR [95%CI] = 1.37 [1.08–1.74], *p* = 0.009), and < 80 mmHg (increase per 1 h) (adjusted OR [95%CI] = 1.35 [1.06–1.73], *p* = 0.016) were independently associated with the occurrence of POD, while after the initiation of microscopic surgery, values of MAP < 70 mmHg (increase per 1 h) (adjusted OR [95%CI] = 1.37 [1.01–1.85], *p* = 0.046), < 75 mmHg (increase per 1 h) (adjusted OR [95%CI] = 1.52 [1.09–2.13], *p* = 0.014), and < 80 mmHg (increase per 1 h) (adjusted OR [95%CI] = 1.47 [1.02–2.1], *p* = 0.037) were independently associated with POD.

## Discussion

### Main results of this study

The mean blood pressure in the delirium group was significantly lower than that in the no delirium group both before and after the initiation of microsurgery. Even after adjusting for delirium-related factors, MAP of < 70, < 75, and < 80 mmHg before the initiation of microsurgery were independently associated with the occurrence of POD. MAP of < 70, < 75, and < 80 mmHg after the initiation of microsurgery were also independently associated with the occurrence of POD.

### Comparison with previous studies

The relationship between duration of IOH and occurrence of POD in non-cardiac surgeries was investigated in two previous studies. One study was a multicenter retrospective observational study involving about 320,000 adult non-cardiac surgery patients, with IOH being defined as MAP below 55 mmHg. The study showed that MAP below 55 mmHg during surgery significantly increased the risk of POD in a time-dependent manner (with the odds ratio of delirium increasing by 1.06 for every 10 min of cumulative time below 55 mmHg) [4]. The other study was a single-center retrospective study involving 605 patients aged 65 years or older undergoing thoracic surgery and orthopedic surgery. IOH was defined as MAP below 65 mmHg, and it was found that continuous periods of hypotension lasting 5 min or more below 65 mmHg were significantly associated with the occurrence of POD (odds ratio of 3.93) [5]. Both studies showed a significant association between duration of IOH and occurrence of POD despite differences in surgical procedures, patient cohorts, and definitions of IOH, suggesting results comparable to those of our study.

There was also a study in which IOH and POD in patients undergoing free flap reconstruction were investigated [10]. In that study, a comparison of the lowest intraoperative MAP value in the delirium and no delirium groups showed that the delirium group had a significantly lower MAP value (60 mmHg vs. 65 mmHg, *p* < 0.001) with a cutoff value of 62.5 mmHg. In our study, the lowest MAP after initiation of microsurgery was also significantly lower in the delirium group (53 mmHg in the delirium group vs. 56 mmHg in the no delirium group, *p* = 0.01). However, in that previous study, the duration of hypotension was not examined blood pressures before and after initiation of the flap grafting procedure.

### Interpretation

This is the first study to investigate the relationship between duration of IOH and POD both before and after the initiation of microsurgery. Two interpretations can be drawn from the findings regarding IOH before and after the initiation of microsurgery and its association with POD.

First, the IOH that was significantly associated with POD in this study was defined at MAP thresholds of 70, 75, and 80 mmHg. These thresholds are higher than those in previous studies. The brain has autoregulatory mechanisms to maintain stable cerebral perfusion. However, a drop in MAP to below the autoregulation range leads to impaired cerebral blood flow and potential brain ischemia [15]. This effect is particularly pronounced in patients with hypertension, as the lower limit of autoregulation is higher, making them more susceptible to cerebral hypoperfusion when MAP decreases [16]. Approximately 60% of head and neck cancer patients have comorbid hypertension [17]. Although there was no significant difference in the prevalence of hypertension between the two groups in our cohort, 60.5% of the patients in the delirium group had hypertension, whereas only 46.9% of the patients in the no delirium group had hypertension. Therefore, the lower MAP observed in the delirium group may have had a more significant impact on cerebral perfusion, potentially explaining why the MAP thresholds associated with POD were higher in this study than in previous studies.

Moreover, MAP < 65 mmHg was not associated with the occurrence of postoperative delirium in the multivariate analysis in this study. The possible reasons for this are as follows. In this study, the percentage of time during which MAP was less than 65 mmHg before the start of microsurgery was 34.8%, and after the start of microsurgery, it was 25.6%. The percentage of time during which the MAP was less than 60 mmHg was even lower. In other words, the duration of MAP < 65 mmHg was short, which may explain why MAP < 65 mmHg was not independently associated with the occurrence of postoperative delirium in the multivariate analysis.

Second, the MAP thresholds significantly associated with POD were similar both before and after the initiation of microsurgery. Although MAP was significantly higher after the initiation of microsurgery than before (before initiation of microsurgery: 70.4 mmHg [standard deviation (SD): 6.4] vs. after initiation of microsurgery: 72.0 mmHg [SD: 6.9], *p* = 0.0000565), the difference was only about 2 mmHg (supplemental Fig. 1). This small difference may explain why the MAP thresholds associated with POD were the same before and after the initiation of microsurgery.

Therefore, maintaining MAP of at least 80 mmHg not only after, but also the initiation of microsurgery may be necessary to prevent POD.

### Limitations

This study has several limitations. First, this study was a single-center retrospective study with a small number of patients in the delirium group, which limits the ability to fully adjust for confounding factors related to POD. Second, the lack of a prior sample size calculation may limit the statistical power and generalizability of our findings. Third, postoperative blood pressure and oxygenation may be related to postoperative delirium in free flap reconstruction. However, because postoperative vital signs were not investigated in this study, the relationship between the study results and postoperative vital signs remains unclear. Future prospective investigation is needed. Fourth, many patients in this study cohort had comorbid hypertension, which may have influenced the results. However, the impact of hypertension is likely to be small if blood pressure is well-controlled with medication, even in patients with hypertension. Fifth, since this study did not investigate blood pressure control, we were unable to accurately assess the relationship between our findings and hypertension. although POD can occur up to 7 days postoperatively, this study only followed patients for 48 h after surgery in the ICU. As a result, patients who might have developed POD after 48 h were not included, which may have led to false-negative cases. Finally, preoperative cognitive function is a crucial risk factor for POD, but formal cognitive assessments, such as the MMSE, were not conducted in this study.

## Conclusion

In this study, patients who developed POD had significantly lower intraoperative MAP. Furthermore, when MAP thresholds were set at 70, 75, and 80 mmHg, prolonged periods below these values before and after the initiation of microsurgery were independently associated with the occurrence of POD. Based on our findings, maintaining MAP of 80 mmHg or higher may help prevent postoperative delirium.

## Supplementary Information

Below is the link to the electronic supplementary material.

Supplementary material 1 (DOCX 16 kb)

## Data Availability

The datasets used and/or analyzed during the current study are available from the corresponding author upon reasonable request.
