# Supplementary material for: Relationship between duration of intraoperative hypotension and postoperative delirium in patients undergoing head and neck cancer surgery with free flap reconstruction: a retrospective observational study
Source: J Anesth. 2025 Jul 13;40(1):20–9. doi: 10.1007/s00540-025-03538-2 (PMC12860842; doi:10.1007/s00540-025-03538-2)
Supplement: Supplementary file 1 — Supplementary material 1 (DOCX 16 kb) [file 540_2025_3538_MOESM1_ESM.docx]

Supplemental table 1 Comparison of hemodynamics before and after initiation of microsurgery

|  | Before initiation of microsurgery | After initiation of microsurgery | *p*-value |
| --- | --- | --- | --- |
| Systolic blood pressure (mmHg) | 107.8 (8.5) | 111.2 (8.7) | <0.001 |
| Mean blood pressure (mmHg) | 70.4 (6.4) | 72.0 (6.9) | <0.001 |
| Diastolic blood pressure (mmHg) | 51.0 (6.2) | 51.8 (6.6) | 0.00566 |
| Heart rate (bpm) | 70.9 (10.3) | 77.5 (11.5) | <0.001 |

The data are presented as average (Standard deviation: SD).
